# Liquid Biopsy in HPV-Associated Head and Neck Cancer: A Comprehensive Review

**DOI:** 10.3390/cancers17060977

**Published:** 2025-03-13

**Authors:** Federica Maria Parisi, Mario Lentini, Carlos M. Chiesa-Estomba, Miguel Mayo-Yanez, Jerome R. Leichen, Matthew White, Giovanni Giurdanella, Salvatore Cocuzza, Maria Rita Bianco, Nicolas Fakhry, Antonino Maniaci

**Affiliations:** 1 Department of Medical and Surgical Sciences and Advanced Technologies “G.F. Ingrassia”, ENT Section, University of Catania, 95125 Catania, Italy; federicamariaparisi@gmail.com (F.M.P.); s.cocuzza@unict.it (S.C.); 2 Department of Otolaryngology, ASP 7, Ragusa Hospital, 97100 Ragusa, Italy; 3 Department of Otorhinolaryngology-Head and Neck Surgery, Hospital Universitario Donostia, 20001 San Sebastian, Spain; 4 Otorhinolaryngology-Head and Neck Surgery Department, Complexo Hospitalario Universitario A Coruña (CHUAC), 15006 La Coruña, Spain; miguelmmy@gmail.com; 5 Otorhinolaryngology-Head and Neck Surgery Department, Hospital San Rafael (HSR) de A Coruña, 15006 La Coruña, Spain; 6 Otorhinolaryngology-Head and Neck Surgery Research Group, Institute of Biomedical Research of A Coruña, (INIBIC), Complexo Hospitalario Universitario de A Corñna (CHUAC), Universidade da Corñna (UDC), 15494 La Coruña, Spain; 7 Department of Human Anatomy and Experimental Oncology, UMONS Research Institute for Health Sciences and Technology, University of Mons (UMons), 7011 Mons, Belgium; jerome.leichen@umons.ac.be; 8 Division of Otorhinolaryngology, Head and Neck Surgery, University of Cape Town, Cape Town 8001, South Africa; matthewwhite86@yahoo.com; 9 Department of Medicine and Surgery, University of Enna “Kore”, 94100 Enna, Italy; giovanni.giurdanella@unikore.it; 10 Otolaryngology-Department of Health Science, University of Catanzaro, 88100 Catanzaro, Italy; mrbianco@unicz.it; 11 Department of Oto-Rhino-Laryngology Head and Neck Surgery, La Conception University Hospital, AP-HM, Aix Marseille Université, 13006 Marseille, France; nicolas.fakhry@ap-hm.fr

**Keywords:** HPV-positive HNSCC, liquid biopsy, ctDNA

## Abstract

**Objectives:** Head and neck squamous cell carcinoma (HNSCC) is the sixth most common cancer globally, with HPV-positive cases emerging as a distinct subtype with unique clinical and molecular characteristics. Current diagnostic methods, including tissue biopsy and imaging, face limitations in terms of invasiveness, static disease assessment, and difficulty in distinguishing recurrence from treatment-related changes. This review aimed to assess the potential of liquid biopsy as a minimally invasive tool for the diagnosis, treatment monitoring, and surveillance of HPV-associated HNSCC. **Methods:** This systematic review analyzed literature from PubMed/MEDLINE, Embase, and Web of Science, focusing on original research and reviews related to liquid biopsy applications in HPV-positive HNSCC. Included studies were evaluated based on the robustness of the study design, clinical relevance, and analytical performance of liquid biopsy technologies. Biomarker types, detection methods, and implementation strategies were assessed to identify advancements and challenges in this field. **Results:** Liquid biopsy technologies, including circulating HPV DNA, ctDNA, and extracellular vesicles, demonstrated high sensitivity (90–95%) and specificity (>98%) in detecting HPV-positive HNSCC. These methods enabled real-time monitoring of tumor dynamics, early detection of recurrence, and insights into treatment resistance. Longitudinal analysis revealed that biomarker clearance during treatment correlates strongly with patient outcomes. **Conclusions:** Liquid biopsy is a transformative diagnostic and monitoring tool for HPV-associated HNSCC, offering minimally invasive, real-time insights into tumor biology. While challenges remain in standardization and clinical implementation, ongoing research and technological innovations hold promise for integrating liquid biopsy into personalized cancer care, ultimately improving patient outcomes.

## Simple Summary

Human papillomavirus (HPV)-associated head and neck cancers are increasingly common and require precise, less invasive methods for detection and monitoring. This review explores liquid biopsy, a revolutionary technique analyzing biomarkers from blood samples, which offers unique benefits for HPV-positive cancers. Unlike traditional tissue biopsies, liquid biopsy provides real-time insights into tumor behavior, treatment response, and disease progression while reducing the need for invasive procedures. By detecting both viral DNA and tumor-specific markers, this method enhances early detection and helps tailor treatment plans. Despite technical and implementation challenges, liquid biopsy holds great promise for improving outcomes and advancing personalized care in HPV-related head and neck cancers. This work highlights the current state, technological advances, and future potential of liquid biopsy in cancer management.

## 1. Introduction

Head and neck squamous cell carcinoma (HNSCC) represents the sixth most common cancer worldwide, with approximately 890,000 new cases and 450,000 deaths reported annually [1]. Over the past three decades, human papillomavirus (HPV) infection has emerged as a significant etiological factor, particularly in oropharyngeal cancers, leading to a distinct disease entity with unique clinical and molecular characteristics [2]. The incidence of HPV-positive HNSCC increased by 225% between 1988 and 2004, with projections suggesting it will surpass cervical cancer as the most common HPV-related malignancy by 2025 [3]. HPV-positive HNSCC demonstrates markedly different biological behavior compared to its HPV-negative counterpart, typically presenting in younger patients with limited tobacco and alcohol exposure [4]. These tumors exhibit superior response to treatment and improved survival outcomes, with 3-year overall survival rates of 82% compared to 57% in HPV-negative disease [5]. The distinct molecular landscape of HPV-positive tumors, characterized by p16 overexpression and relatively few mutations, has led to the development of de-escalation protocols aimed at reducing treatment-related toxicity while maintaining oncological efficacy [6]. Despite the significant prognostic and therapeutic implications of HPV status, existing diagnostic methods have several limitations. The gold standard for HPV detection involves tissue biopsy with p16 immunohistochemistry and HPV DNA in situ hybridization [7]. However, this approach requires invasive procedures, may not capture tumor heterogeneity, and provides only a static snapshot of disease status [8]. Furthermore, conventional imaging modalities often struggle to differentiate post-treatment changes from disease recurrence, leading to diagnostic uncertainty and potential delays in intervention [9].

The emergence of liquid biopsy represents a paradigm shift in cancer diagnostics, offering a minimally invasive means to assess tumor biology through the analysis of blood-based biomarkers [10]. This approach holds particular promise in HPV-associated HNSCC, where viral markers provide additional specificity for disease monitoring [11]. Liquid biopsy enables real-time assessment of tumor dynamics, therapy response, and disease progression through the detection and analysis of circulating tumor DNA (ctDNA), circulating tumor cells (CTCs), extracellular vesicles, and cell-free HPV DNA [12]. Recent technological advances have significantly improved the sensitivity and specificity of liquid biopsy techniques, with next-generation sequencing platforms capable of detecting tumor-derived DNA fragments at concentrations as low as 0.1% [13]. This enhanced analytical capability has expanded the potential applications of liquid biopsy across the cancer care continuum, from early detection to treatment monitoring and surveillance [14]. In HPV-positive HNSCC, liquid biopsy offers several unique advantages. The presence of viral DNA sequences provides highly specific markers for disease detection and monitoring, potentially enabling earlier identification of recurrence compared to conventional imaging [15]. Additionally, longitudinal analysis of circulating biomarkers may offer insights into treatment response and resistance mechanisms, facilitating more personalized therapeutic approaches [16]. The clinical integration of liquid biopsy in HPV-associated HNSCC management faces several challenges, including standardization of pre-analytical variables, optimization of detection methods, and validation in large prospective cohorts [17]. However, the potential benefits of this approach—including improved disease monitoring, reduced reliance on invasive procedures, and more timely therapeutic interventions—warrant thorough investigation [18]. This comprehensive review aims to examine the current state of liquid biopsy in HPV-associated head and neck cancer, addressing biological foundations, technical considerations, clinical applications, and future directions. We will explore the various biomarker types, analytical platforms, and implementation strategies while critically evaluating the evidence supporting their use in clinical practice. Understanding these aspects is crucial for optimizing the potential of liquid biopsy to improve patient outcomes in HPV-associated HNSCC.

## 2. Materials and Methods

### 2.1. Literature Search Strategy

This review was developed according to a literature screening for retrieving relevant studies reporting liquid biopsy applications in HNSCC related to HPV. We performed literature research across PubMed/MEDLINE, Embase, and Web of Science. The search included Medical Subject Headings (MeSH) and free-text terms pertaining to liquid biopsy techniques (circulating tumor DNA, droplet digital PCR, and next-generation sequencing), HPV-associated head and neck cancer, and diagnostic and prognostic applications of ctDNA. Manual searches were also employed in the reference lists of pertinent studies and reviews to ensure systematic coverage.

### 2.2. Eligibility and Exclusion Criteria

We included original research articles, systematic reviews, or meta-analyses assessing different liquid biopsy techniques in HPV-positive HNSCC for study selection. We selected studies if they evaluated clinical utility (diagnostic accuracy, treatment response monitoring, recurrence detection); utilized validated detection technologies (e.g., qPCR, ddPCR, NGS, hybrid capture sequencing); reported relevant clinical outcomes (e.g., sensitivity, specificity, overall survival, progression-free survival); and were published in English. We excluded case reports, editorials, commentaries, and conference abstracts without complete data, studies strictly on HPV-negative HNSCC, studies only assessing non-blood-based liquid biopsy, animal studies, and in vitro studies without clinical associations. Two independent reviewers screened the articles using these criteria, and any discrepancies were resolved by discussion or consultation with a third senior reviewer.

### 2.3. Study Quality Assessment

Standardized quality assessment tools were utilized to enforce the rigor and reliability of studies added to the review. The Newcastle–Ottawa Scale was applied to observational cohorts and case-control studies, examining the indicators of selection bias, comparability, and reporting of outcomes. For diagnostic accuracy studies, we assessed the risk of bias in the areas of patient selection, index test result, reference standard, and flow of patients in accordance with the QUADAS-2 tool. Each study was independently scored by two reviewers, and studies with low-quality scores were excluded from final synthesis.

### 2.4. Data Extraction and Synthesis

We extracted data from the included studies on the study design and population characteristics (sample size and methods of confirmation of HPV status); liquid biopsy methodology (sample processing and ctDNA detection methods, target markers assessed, sequencing platforms used); clinical outcomes (sensitivity, specificity, and predictive value for detection of recurrence); and implementation considerations (laboratory workflow, cost, and turnaround time). Due to the significant heterogeneity in methods, patient populations, and outcomes measured in the included studies, a formal meta-analysis was not conducted. The findings were instead synthesized for a narrative review, organizing studies by liquid biopsy technology, clinical application, and methodological quality.

## 3. Results

### 3.1. Biology of HPV-Associated Head and Neck Cancer

The oncogenic transformation in HPV-associated head and neck cancer primarily involves high-risk HPV types, with HPV-16 accounting for approximately 90% of cases [19]. The carcinogenic process begins with viral entry into the basal cells of the epithelium, followed by the integration of viral DNA into the host genome. This integration represents a critical step in malignant transformation, disrupting normal cell cycle regulation through the expression of viral oncoproteins E6 and E7 [20]. E6 protein promotes the degradation of tumor suppressor p53, while E7 inactivates the retinoblastoma protein (pRb), leading to cell cycle dysregulation and genomic instability [21]. This mechanism differs significantly from the carcinogenesis of HPV-negative tumors, which typically arise from the progressive accumulation of mutations due to environmental factors such as tobacco and alcohol exposure [22]. HPV-positive head and neck cancers exhibit unique molecular characteristics that distinguish them from their HPV-negative counterparts. These tumors typically display wild-type p53, elevated expression of p16INK4a (a surrogate marker for HPV infection), and fewer overall mutations [23]. Genome-wide analyses have revealed distinct mutational landscapes, with HPV-positive tumors showing fewer copy number alterations and a lower frequency of mutations in TP53 and CDKN2A genes [24]. Recent studies using next-generation sequencing have identified specific molecular signatures, including unique DNA methylation patterns and microRNA expression profiles that correlate with improved prognosis in HPV-positive disease [25]. The molecular distinction extends to altered signaling pathways, with HPV-positive tumors showing increased activation of the PI3K/AKT/mTOR pathway and cell cycle regulators [26]. These tumors also demonstrate unique patterns of DNA damage repair pathway activation, which may explain their enhanced. Cumulative evidence demonstrates that HPV-associated HNSCC has greater sensitivity to radiotherapy than HPV-negative tumors, a characteristic that has spurred attempts to de-escalate treatment in appropriate patient subsets [27]. Nonetheless, we need to differentiate radiosensitivity from the long-term efficacy of treatment. SEER data suggest that although HPV+ patients may demonstrate improved early local tumor regression in response to RT, long-term outcomes do not improve with RT monotherapy. Rather, favorable prognosis with attention to multimodal therapy is the mainstay, coupled with the notable survival benefit with CRT. Additionally, while HPV16 status is regarded as a prognostic biomarker, its influence is treatment-dependent and it may also act as a predictive biomarker, affecting response rates to the therapeutic strategy applied. This highlights a necessity for individualized treatment planning, which considers not only the biological radiosensitivity of HPV+ tumors but also the established advantages of CRT for long-term disease control. Notably, HPV-positive tumors express viral antigens that serve as potential targets for immune recognition and therapeutic intervention [28]. The tumor microenvironment (TME) of HPV-positive head and neck cancers exhibits distinct immunological features that contribute to their improved prognosis. These tumors typically show higher levels of tumor-infiltrating lymphocytes (TILs), particularly CD8+ T-cells, indicating a more robust anti-tumor immune response [29]. The presence of these immune cells correlates with better treatment outcomes and survival rates compared to HPV-negative disease [30]. The immune landscape in HPV-positive tumors is characterized by increased expression of immune checkpoint molecules, including PD-1/PD-L1, suggesting potential vulnerability to immune checkpoint inhibition [31]. Recent studies have demonstrated differential expression of inflammatory mediators and cytokines between HPV-positive and -negative tumors, with HPV-positive cases showing enhanced type I interferon responses and T-helper 1 (Th1) cell signatures [32].

The stromal composition also differs significantly, with HPV-positive tumors typically showing altered extracellular matrix organization and distinct patterns of cancer-associated fibroblast activation [33]. These tumors demonstrate unique angiogenic profiles, with different patterns of vascular endothelial growth factor (VEGF) expression and microvessel density compared to HPV-negative cases [34].

Understanding these biological differences has profound implications for therapeutic approaches. The distinct molecular and immunological features of HPV-positive tumors have led to the development of targeted therapies and immunotherapeutic strategies specifically designed for this patient population [35]. For instance, the enhanced immune recognition of viral antigens has prompted investigations into therapeutic vaccines and adoptive T-cell therapies [36].

Recent advances in single-cell sequencing and spatial transcriptomics have provided deeper insights into tumor heterogeneity and evolution in HPV-positive disease. These studies have revealed complex interactions between tumor cells and the immune microenvironment, identifying potential therapeutic targets and resistance mechanisms [37]. The temporal dynamics of HPV-driven carcinogenesis, particularly the role of persistent viral infection and immune evasion strategies, continue to be areas of active investigation [38].

Understanding these biological aspects is crucial for the development and optimization of liquid biopsy approaches, as the distinct molecular and immunological features of HPV-positive tumors provide unique opportunities for biomarker development and monitoring strategies. The presence of viral DNA and associated molecular changes offers specific targets for detection and quantification in liquid biopsy specimens, potentially enabling more sensitive and specific disease monitoring compared to HPV-negative cases [39].

### 3.2. Liquid Biopsy Fundamentals

The reliability of liquid biopsy biomarkers in HPV-positive head and neck cancer varies depending on the specific analyte and detection method employed. Circulating tumor DNA (ctDNA) has emerged as one of the most promising biomarkers, with studies demonstrating detection rates of 90–95% in advanced disease [40]. HPV-associated head and neck squamous cell carcinoma (HNSCC) is biologically and clinically distinct from its HPV-negative counterpart, with unique tumor behavior and biomarker profiles that influence liquid biopsy performance. HPV+ HNSCC primarily arises in the oropharynx and is associated with a strong immune response and better overall survival compared to HPV-negative cases. These tumors exhibit higher levels of circulating tumor DNA (ctDNA) due to their distinct viral etiology, making HPV ctDNA a particularly sensitive biomarker for disease monitoring. However, while ctDNA is a valuable tool, circulating tumor cells (CTCs) detection presents a challenge in HPV+ cases, as these tumors tend to have lower epithelial–mesenchymal transition (EMT) properties, which may reduce the number of CTCs available for analysis. Additionally, extracellular vesicles (EVs) released by HPV+ tumors contain both viral and tumor-derived components, suggesting a potential role in tracking disease progression and immune modulation. Understanding these biological particularities is crucial for selecting the most effective liquid biopsy technique, ensuring that each approach aligns with the molecular characteristics of HPV+ HNSCC. The presence of viral DNA sequences provides an additional layer of specificity, as HPV16 DNA can be detected in plasma with high sensitivity using digital PCR techniques [41]. While the majority of studies on liquid biopsy in HPV-associated head and neck squamous cell carcinomas (HNSCCs) have been conducted with a focus on oropharyngeal (OP) cancers, HPV positivity also occurs in non-oropharyngeal sites such as the larynx, hypopharynx, and oral cavity. The clinical behavior and biomarker profile of non-OP HPV+ HNSCC are distinct from OP cases, and these differences can result in the variability of ctDNA shedding and ctDNA detection rates. Though circulating HPV DNA assays have shown high sensitivity (90–95%) and specificity (>98%) in detection of oropharyngeal cancer, their clinical applicability to non-OP HPV+ cases is less well studied. This distinction is important with respect to liquid biopsy for surveillance and therapeutic monitoring, as non-OP HPV+ cases may not share biomarker dynamics observed in OP cancers.

#### 3.2.1. Comparative Analysis of ctDNA Detection Methods

The evaluation of ctDNA in HPV-associated head and neck squamous cell carcinoma (HNSCC) relies on multiple methodological approaches, each with distinct advantages and limitations. The most commonly employed techniques include quantitative polymerase chain reaction (qPCR), droplet digital PCR (ddPCR), and next-generation sequencing (NGS) (Table 1).

**Figure 1:**
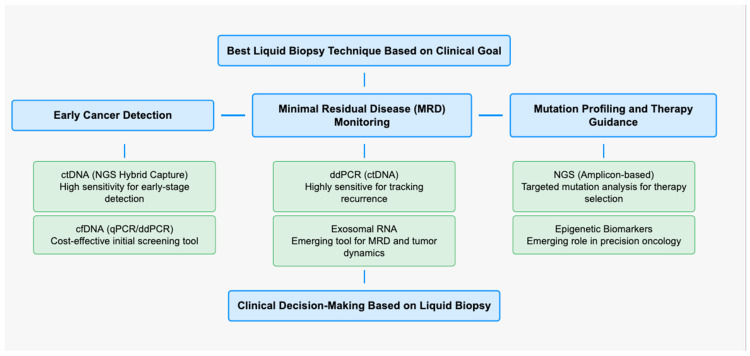
Conceptual map showing the difference in liquid biopsy technique features, depending on the clinical or diagnostic goal.

Recent studies have demonstrated that ddPCR achieves a pooled sensitivity of 96% (95% CI: 92–99%) and specificity of 98% (95% CI: 96–99%) for detecting circulating HPV DNA in plasma, making it one of the most reliable techniques for real-time monitoring of HPV-associated HNSCC. qPCR is widely available and cost-effective but has limited sensitivity for detecting low levels of ctDNA. ddPCR improves upon qPCR by offering absolute quantification and higher sensitivity, making it a preferred method for minimal residual disease detection. NGS, including hybrid capture and amplicon-based sequencing, provides comprehensive genomic profiling, allowing for the simultaneous detection of viral and somatic mutations; however, it is associated with higher costs and longer turnaround times. Among these methods, ddPCR is particularly suited for HPV-related HNSCC due to its ability to detect circulating HPV DNA with high specificity and sensitivity (>95%) (Figure 1).

#### 3.2.2. ctDNA Detection, Cost-Effectiveness, and Clinical Utilities for HPV-Associated HNSCC

It is cost-effective for routine clinical monitoring and has demonstrated clinical utility in assessing treatment response. Conversely, NGS offers the advantage of detecting tumor heterogeneity and treatment resistance mechanisms but remains costly and technically complex. Hybrid capture sequencing, though highly sensitive, requires substantial bioinformatics expertise, making it less practical for widespread clinical implementation. The selection of the optimal method depends on the clinical context, with ddPCR emerging as the most viable option for real-time disease monitoring, while NGS is reserved for broader molecular characterization. ctDNA analysis offers unique advantages in HPV-positive disease due to the presence of both human and viral DNA markers. Studies have shown that the quantification of plasma HPV16 DNA correlates significantly with tumor burden and treatment response [42]. While HPV16 is responsible for >90% of HPV-positive HNSCC, other high-risk HPV types including HPV18, HPV31, and HPV33 are seen at low frequencies. There are differences in genotypes for these genotypes, especially in E6 and E7 oncogenes, which are essential for a robust performance of liquid biopsy tests. All of the regularly used ctDNA detection methods are standardized for HPV16, and they sacrifice sensitivity and specificity in detecting non-HPV16 genotypes (dPCR and NGS). Many of the differences including alterations in the sites of viral integration (etc.) in these types out of the non-HPV16 type as well as polymorphisms of succession may have an impact on the requirement for binding and detection range of the used test that represent the need of more thorough investigation for providing correct identification of clades in a greater variety of subtypes of HPVs [6]. Future studies should continue to explore methods of assay optimization to increase the clinical usefulness of liquid biopsy in the diagnosis of non-HPV16 HPV-associated HNSCC. The detection of both viral and somatic mutations in ctDNA provides complementary information about disease status, with some studies reporting lead times of several months compared to conventional imaging for recurrence detection [15]. Advanced sequencing techniques have enabled the identification of tumor-specific mutations and viral integration sites, offering insights into disease evolution and treatment resistance [43]. Circulating tumor cells provide valuable information about metastatic potential and tumor heterogeneity in HPV-positive head and neck cancer. Novel CTC isolation techniques have demonstrated the ability to capture cells expressing viral oncoproteins, enabling direct assessment of HPV status and tumor cell characteristics [44]. The molecular analysis of CTCs has revealed distinct phenotypes associated with treatment resistance and metastatic potential, although technical challenges in isolation and characterization remain [45]. Extracellular vesicles (EVs) represent an emerging biomarker class, carrying tumor-derived proteins, nucleic acids, and viral components. Studies have shown that EVs from HPV-positive tumors contain specific microRNA signatures and viral transcripts that could serve as diagnostic markers [46]. The analysis of EV cargo has revealed novel mechanisms of tumor–host interaction and potential therapeutic targets [47].

Cell-free HPV DNA represents a highly specific biomarker for HPV-positive disease. Recent studies have demonstrated its utility in early detection and disease monitoring, with sensitivity rates exceeding 95% in some cohorts [48]. The quantitative assessment of plasma HPV DNA has shown promise in predicting treatment response and detecting minimal residual disease [49]. Standardization of pre-analytical variables is crucial for reliable liquid biopsy results. Studies have demonstrated that specialized blood collection tubes containing preservatives can maintain sample integrity for up to 7 days [50]. Optimal processing protocols include rapid plasma separation (within 4 h of collection) and standardized DNA extraction methods to maximize yield and quality [51]. The timing of sample collection relative to treatment has also been shown to impact biomarker detection, with certain time points providing more informative results [52]. Next-generation sequencing platforms have demonstrated superior sensitivity for detecting low-frequency variants and viral sequences [53]. Digital PCR technologies offer advantages in the quantitative assessment of specific markers, particularly for monitoring treatment response [54]. Novel platforms combining multiple detection modalities have shown promise in comprehensive biomarker analysis, enabling simultaneous assessment of different analyte types [55]. The integration of multiple biomarker types through pan-omic approaches has emerged as a powerful strategy for comprehensive disease monitoring. Studies utilizing combined analysis of ctDNA, CTCs, and EVs have demonstrated improved sensitivity and specificity compared to single-marker approaches [56]. Advanced computational methods and artificial intelligence algorithms have enhanced the interpretation of complex liquid biopsy data, enabling more accurate disease monitoring and prediction of outcomes [57]. Recent technological developments have focused on improving detection sensitivity and specificity through novel molecular barcoding strategies and enhanced isolation methods [58]. The application of machine learning algorithms to liquid biopsy data has facilitated the identification of complex biomarker patterns associated with disease progression and treatment response [59]. Although this review does not constitute a formal meta-analysis, it aspires to a comprehensive comparative assessment of liquid biopsy methods and the consequences of these for various clinical scenarios. Technological approaches to the assessment of circulating tumor DNA (ctDNA) in HPV-associated head and neck squamous cell carcinoma (HNSCC) differ in sensitivity, specificity, and clinical utility.

### 3.3. Applications in Clinical Practice

**Figure 2:**
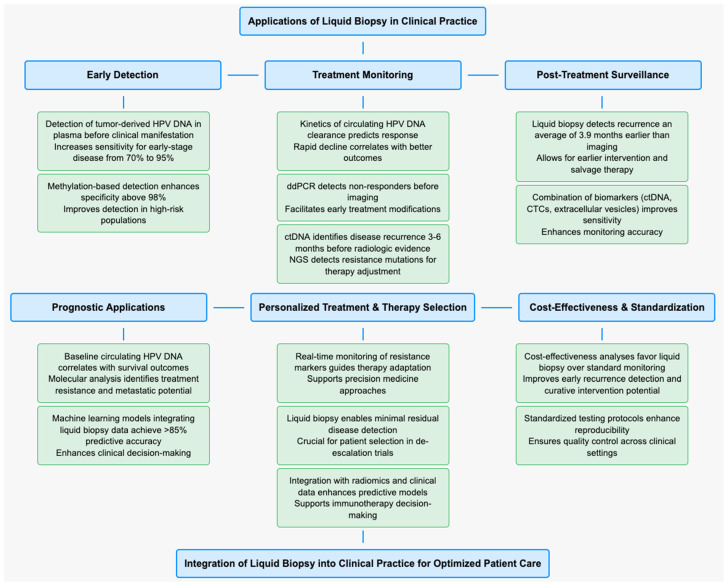
Conceptual map describing the different applications of liquid biopsy in clinical practice.

Early detection of HPV-associated head and neck cancer through liquid biopsy represents a promising application with potential to improve patient outcomes. Studies have demonstrated the ability to detect tumor-derived HPV DNA in plasma months before clinical manifestation of disease [60]. A prospective cohort study showed that combining plasma HPV DNA detection with traditional screening methods increased sensitivity for early-stage disease from 70% to 95% [61]. Novel approaches utilizing methylation patterns of circulating DNA have further enhanced early detection capabilities, with specificity rates exceeding 98% in high-risk populations [62]. As a dynamic probe of tumor evolution, liquid biopsy technology is providing critical information on treatment efficacy and disease recurrence in-characteristics that are key for transferring their benefits into the management of patients with cancer. The kinetics of circulating HPV DNA clearance during therapy is a robust predictor of response, with rapid decline correlating with improved outcomes. ddPCR-based monitoring has been shown in studies to detect non-responders earlier than imaging and facilitates prompt treatment modifications. Compared with the standard method, liquid biopsy can detect disease recurrence 3–6 months ahead of the appearance of radiologic evidence as ctDNA can been found in plasma before radiologic evidence of disease recurrence. ddPCR is well poised to detect early recurrence due to a rapid turnaround time and quantitation of minimal residual disease. In comparison, broad genomic profiling approaches (NGS-based methods) deliver more molecular information that can recognize resistance mutations that influence the choice of second-line treatment. By incorporating this knowledge, clinicians can tailor therapy, minimize unwarranted measures, and optimize the clinical course (Figure 2).

Liquid biopsy has emerged as a reliable method for determining HPV status, offering advantages over traditional tissue-based testing. Jakobsen et al. reported a 100% concordance between HPV genotype in tumor tissue and plasma in patients with HPV-positive oropharyngeal squamous cell carcinoma. The baseline sensitivity for circulating cell-free HPV DNA detection was 97.2% (95% CI: 90.3–99.6) [61]. The ability to detect specific HPV genotypes and viral integration patterns through circulating biomarkers provides additional prognostic information. Digital PCR-based approaches have shown particular promise, with detection limits as low as 0.01% variant allele frequency [63].

However, while liquid biopsy techniques provide predictive value for treatment response, their role in prognosis depends on additional clinical factors such as treatment regimen and tumor biology. Treatment monitoring represents one of the most validated applications of liquid biopsy in HPV-positive head and neck cancer. Longitudinal studies have shown that changes in circulating HPV DNA levels correlate strongly with treatment response, with dramatic decreases observed within the first few weeks of effective therapy [64,65]. Integration of liquid biopsy data with imaging results has improved response assessment accuracy, enabling earlier identification of treatment failure [66].

Post-treatment surveillance using liquid biopsy has demonstrated significant advantages over conventional monitoring approaches. Studies have shown that rises in circulating tumor DNA can detect recurrence an average of 3.9 months earlier than standard imaging [67]. Implementation of structured surveillance protocols incorporating regular liquid biopsy testing has led to earlier intervention in cases of recurrence, potentially improving salvage treatment outcomes [68]. The combination of multiple biomarker types, including circulating tumor cells and extracellular vesicles, has further enhanced surveillance sensitivity [69].

Prognostic applications of liquid biopsy have shown considerable promise in stratifying patient risk and predicting outcomes. Baseline levels of circulating HPV DNA have demonstrated strong correlation with overall survival and progression-free survival [70]. Molecular analysis of circulating biomarkers has revealed distinct patterns associated with treatment resistance and metastatic potential [48]. Machine learning approaches integrating multiple liquid biopsy parameters have achieved predictive accuracies exceeding 85% for major clinical outcomes [71]. HPV ctDNA is a valuable OP-HNSCC biomarker, but the prognostic and diagnostic value of HPV ctDNA in non-oropharyngeal HPV+ tumors is not as clearly defined. HPV-induced tumors beyond the oropharynx possess specific shedding patterns of ctDNA, which can be due to costaining viral burdens or diverse tumor–host interactions. A lack of studies on liquid biopsy application to non-OP HPV+ HNSCC points to the need for further studies. The clinical utility of liquid biopsy extends beyond traditional applications, with emerging evidence supporting its role in treatment selection and modification. Studies have shown that real-time monitoring of molecular resistance markers can guide therapy adaptation, potentially improving treatment outcomes [72]. The ability to detect minimal residual disease has particular importance in de-escalation trials, where careful patient selection is crucial [73]. Recent technological advances have enabled more sophisticated applications, including the detection of specific resistance mutations and immune response markers [74]. The integration of liquid biopsy data with radiomics and clinical parameters has created powerful predictive models for personalized treatment approaches [75]. Emerging evidence suggests that liquid biopsy can also provide insights into immune checkpoint inhibitor response, potentially guiding immunotherapy decisions [76].

Cost-effectiveness analyses have demonstrated favorable outcomes for liquid biopsy-based surveillance compared to conventional monitoring strategies, particularly when considering earlier detection of recurrence and potential for curative intervention. The implementation of standardized testing protocols and quality control measures has improved the reliability and reproducibility of liquid biopsy results across different clinical settings [77].

### 3.4. Technical Challenges

The analytical performance of liquid biopsy in HPV-associated head and neck cancer is influenced by multiple technical factors. Pre-analytical variables, including blood collection methods, processing time, and storage conditions, significantly impact detection sensitivity [78]. Studies have shown that plasma yield and DNA quality vary substantially based on collection tube type, with specialized cell-free DNA tubes demonstrating superior preservation compared to standard EDTA tubes [79]. Temperature fluctuations during sample transport and storage can affect molecular integrity, with optimal results achieved when samples are processed within 6 h of collection [80].

Molecular detection methods present their own challenges, with different platforms showing varying sensitivity levels. Digital PCR approaches typically demonstrate lower detection limits (0.1% allele frequency) compared to traditional qPCR methods but may miss complex genomic alterations [81]. Next-generation sequencing offers comprehensive genomic profiling but requires sophisticated bioinformatics pipelines to distinguish true variants from artifacts, particularly in cases with a low tumor fraction [13].

Sample stability optimization remains crucial for reliable liquid biopsy results. Recent studies have identified critical factors affecting biomolecule preservation, including the role of nuclease inhibitors and stabilizing agents [82]. Novel preservation methods, such as microfluidic-based approaches, have shown promise in maintaining sample integrity during extended storage periods [83]. The development of standardized protocols for sample handling has significantly improved reproducibility across different laboratory settings [84].

Standardization challenges encompass multiple aspects of the liquid biopsy workflow. Inter-laboratory variability in extraction methods, analytical platforms, and reporting criteria has complicated result interpretation and clinical implementation [85]. The lack of universal reference materials for HPV-positive disease has hindered assay validation and quality control efforts [86]. Technical variation in sequencing approaches, including library preparation methods and bioinformatics pipelines, contributes to result heterogeneity [87].

Cost considerations remain a significant factor in the widespread adoption of liquid biopsy testing. Comprehensive economic analyses have evaluated the cost-effectiveness of different testing strategies, considering factors such as frequency of monitoring and clinical utility. While initial testing costs may be higher compared to conventional methods, studies suggest potential cost savings through earlier detection of recurrence and more efficient treatment monitoring [88].

The technical landscape continues to evolve with emerging technologies addressing current limitations. Novel enrichment methods, including selective capture approaches and molecular barcoding strategies, have improved detection sensitivity for low-abundance variants [89]. Advanced computational methods, incorporating machine learning algorithms, have enhanced the ability to distinguish true signals from technical artifacts [90].

Quality control measures have become increasingly sophisticated, with the development of synthetic controls and standardized reference materials [91]. International initiatives have established guidelines for analytical validation and reporting, although implementation varies across jurisdictions [92]. The integration of multiple quality metrics, including molecular and technical controls, has improved result reliability and reproducibility [93].

Technical advances in sample processing have led to automated systems that reduce manual handling and potential sources of error. These platforms incorporate standardized protocols for extraction, library preparation, and analysis, improving workflow efficiency and result consistency [94]. The development of point-of-care testing platforms shows promise for expanding access to liquid biopsy testing, particularly in resource-limited settings [95].

The field continues to address technical challenges through innovative approaches and collaborative efforts. Multi-institutional studies have helped establish best practices for sample handling and analysis, while technological advances continue to improve sensitivity and specificity [14]. The emergence of integrated testing platforms, combining multiple biomarker analyses, offers potential solutions to current technical limitations while maintaining cost-effectiveness [40].

### 3.5. Implementation Considerations

The implementation of liquid biopsy in clinical practice for HPV-associated head and neck cancer is supported by growing evidence across multiple domains. Prospective clinical validation studies have demonstrated concordance rates exceeding 90% between tissue- and plasma-based HPV detection methods [96]. Large-scale multicenter trials have established the clinical validity of liquid biopsy for treatment monitoring, with significant correlations between circulating biomarker levels and patient outcomes [97]. Cost-effectiveness continues to be an important consideration for the implementation of ctDNA testing in clinical practice. Previous comparative analyses have shown ddPCR to have a better cost–benefit ratio, owing to their lower operational costs than NGS-based approaches. A modeling study estimated that introduction of ddPCR-based surveillance provided better health outcomes at lower (or comparable) costs, as detection of recurrence could be made earlier, leading to better treatment and reduced utilization of repeated imaging and invasive biopsies. Moreover, evidence points to improved patient outcomes with treatment adaptation based on the liquid biopsy, reducing unnecessary chemotherapy or radiation without compromising on patient care. Although NGS is the mainstay of high-throughput molecular insight, its cost grounds it from widespread use. However, due to the resource-intensive process of sequencing and the requirement for specialized bioinformatics interpretation, it poses a financial barrier in these settings, especially in resource-constrained environments. An emerging cost-effective strategy would be hybrid models combining ddPCR for routine monitoring and reserving NGS for cases of suspected treatment resistance or recurrence. Future work should include the establishment of reimbursement approaches that will incentivize the adoption of ctDNA testing in usual care but still allow appropriate access for patients.

Meta-analyses incorporating data from over 2000 patients have confirmed the prognostic value of liquid biopsy results, particularly for early detection of recurrence [98].

Integration of liquid biopsy into existing clinical pathways requires careful consideration of workflow optimization and resource allocation. Studies examining implementation strategies have identified key decision points for testing, including optimal timing relative to treatment and frequency of monitoring [77]. Successful integration models have demonstrated the importance of multidisciplinary coordination, with clear communication channels between clinicians, laboratory staff, and pathologists [99]. Healthcare systems have developed structured protocols for sample collection, processing, and reporting, ensuring consistent test utilization and result interpretation [100].

The rapid evolution of liquid biopsy technology necessitates regular updates to clinical practice guidelines. Professional organizations have begun incorporating liquid biopsy recommendations into their standard-of-care documents, particularly for treatment monitoring and surveillance [101]. Expert panels have proposed specific criteria for test ordering, result interpretation, and clinical decision-making based on liquid biopsy findings [102]. Guidelines addressing quality assurance, laboratory accreditation, and proficiency testing requirements are being developed to ensure reliable test performance [103].

Regulatory considerations present significant challenges for widespread implementation. Current regulatory frameworks vary across jurisdictions, affecting test validation requirements and clinical applications [104]. Laboratory accreditation bodies have established specific criteria for liquid biopsy testing, including personnel qualifications, quality control measures, and proficiency testing programs [105].

The implementation landscape is further shaped by practical considerations regarding laboratory infrastructure and personnel requirements. Successful programs have invested in dedicated facilities and specialized training programs to ensure technical proficiency [106]. Quality management systems incorporating regular monitoring and performance assessment have proven essential for maintaining testing standards [107].

Data management and reporting systems play crucial roles in successful implementation. Electronic health record integration has facilitated result communication and clinical decision support [108]. Standardized reporting formats have improved result interpretation and comparison across institutions [109]. Security measures protecting patient privacy and genetic information have been enhanced to address specific concerns related to molecular testing [110].

Cost considerations and resource allocation continue to influence implementation decisions. Economic analyses have evaluated the impact of different testing strategies on healthcare resources [111]. Novel funding models and risk-sharing arrangements have emerged to support test implementation while managing costs [112]. The development of value-based care metrics has helped justify investment in liquid biopsy infrastructure [113].

The adoption of liquid biopsy technologies into clinical practice necessitates careful infrastructure planning that is fundamentally different than tissue biopsy diagnostics. HPV-associated head and neck squamous cell carcinoma (HNSCC) needs to be testing laboratories have reliable, cross-phase comprehensive systems to control reliability and accuracy. The integrity of a sample is essential in the pre-analytical phase. Blood collection and processing procedures should be standardized as much as possible, and samples should ideally be processed within four hours of collection or stored in a validated way. Specifically, processing whole blood samples using specialized cfDNA stabilization tubes, such as those from Streck, PAXgene, and others, is critical for avoiding degradation that could hinder the recovery of ctDNA and its subsequent analysis. This press release is not an invitation to buy shares in the company. Droplet digital PCR systems such as Bio-Rad QX200 or Stilla Naica are already available and are relatively cheap for clinical applications, while the NGS approaches need more significant investments in high-throughput sequencing machines and bioinformatics resources. Each technology has unique strengths regarding sensitivity, specificity, and throughput that laboratories must balance against clinical requirements and resource availability. The post-analytical dimension focuses on data interpretation capabilities and systems, in particular for NGS-based assays, which rely heavily on complex bioinformatics pipelines for precise variant calling and accurate identification of HPV integration sites. Also, systematic application of quality control through synthetic spike-in controls and standardized reference materials will be needed for consistent validation of the assay. The initial building block of clinical liquid biopsy is regulatory compliance. Laboratories are required to comply with standards such as CLIA, CAP, etc. in the USA whereas ISO 15189 is followed in operations outside the USA. These guidelines permit the necessary validation, reproducibility, and quality control for the complete testing procedure.

Educational initiatives supporting implementation have targeted multiple stakeholder groups. Training programs for healthcare providers have improved their understanding of test capabilities and limitations [114]. Patient education materials have enhanced informed decision-making regarding liquid biopsy testing [115]. Continuing education requirements have been established to maintain competency in this rapidly evolving field [116].

### 3.6. Future Perspectives

Emerging technologies promise to revolutionize liquid biopsy applications in HPV-associated head and neck cancer. Novel sequencing platforms incorporating molecular barcoding and error-correction algorithms have demonstrated unprecedented sensitivity for detecting rare variants [117]. Advanced microfluidic devices enable simultaneous analysis of multiple biomarker types, including circulating tumor cells, cell-free DNA, and extracellular vesicles [118]. Artificial intelligence-driven approaches are improving signal detection and result interpretation, with machine learning algorithms showing superior accuracy in identifying clinically relevant patterns [119].

The integration of artificial intelligence into liquid biopsy analysis represents a significant advancement. Deep learning algorithms have demonstrated superior performance in analyzing complex molecular data and identifying subtle patterns that may indicate disease progression or treatment resistance [120]. Natural language processing techniques are improving the extraction of clinically relevant information from test reports and medical records [121]. Predictive models incorporating multiple data streams have shown promise in personalizing treatment decisions based on liquid biopsy results [122].

Investigation of novel biomarkers continues to expand the utility of liquid biopsy. Studies of circulating tumor-derived metabolites have revealed new opportunities for disease monitoring [123]. Epigenetic markers, including methylation patterns and nucleosome positioning, provide additional layers of diagnostic information [124]. Novel approaches to analyzing tumor-derived extracellular vesicles have identified promising biomarker candidates for early detection and monitoring [125].

The clinical applications of liquid biopsy are expected to expand significantly. Integration with other diagnostic modalities, including imaging and molecular pathology, will enable more comprehensive disease assessment [126]. Point-of-care testing platforms under development may facilitate rapid decision-making in clinical settings [127]. Novel applications in screening and prevention programs show promise for improving early detection rates [128].

The future of liquid biopsy will likely see increased personalization of testing strategies. Advanced computational methods are enabling real-time adjustment of monitoring protocols based on individual patient characteristics and response patterns [48]. The development of therapeutic monitoring platforms that combine drug-level measurements with molecular response markers may optimize treatment delivery [71].

Emerging applications in immunotherapy monitoring represent a particularly promising area. Novel approaches to analyzing circulating immune cells and molecular markers of immune response are improving the prediction of immunotherapy outcomes [129]. Integration of tumor and immune cell analyses may enable a more precise selection of immunotherapy candidates [130].

Technical innovations continue to address current limitations. The development of standardized reference materials and quality control methods will improve result reproducibility across laboratories [131]. Novel sample preparation techniques are enhancing the recovery of rare molecular species [132]. Advanced multiplexing approaches enable simultaneous analysis of multiple biomarker types from limited sample volumes [133].

The field is moving toward more integrated diagnostic approaches. The combination of liquid biopsy results with other molecular and clinical data streams is enabling more sophisticated prognostic models [134]. The development of decision support tools incorporating real-time liquid biopsy data may facilitate more dynamic treatment adjustment [14]. The evolution of regulatory frameworks and reimbursement policies will likely expand access to these advanced diagnostic capabilities [40].

## 4. Conclusions

Liquid biopsy represents a transformative technology in HPV-associated head and neck cancer management and has several unique advantages for diagnosis, monitoring, and surveillance. Of the various ctDNA detection technologies available, ddPCR has shown the most clinically relevant sensitivity, specificity, and cost-effectiveness for routine clinical practice. Although NGS is rich with molecular information, the high cost and complexity limit its use to select cases in need of a detailed genomic interrogation. Therefore, ddPCR-based monitoring should be included in our surveillance strategies since its utility in improving early detection of recurrence and reduced healthcare costs has been previously demonstrated. Optimization of hybrid approaches that best home in on clinical benefit while remaining cost-effective will likely be crucial to ensuring wide access to liquid biopsy technologies at all levels of HPV-associated HNSCC care. The distinct biological characteristics of HPV-positive head and neck cancer, including the presence of viral DNA and specific molecular alterations, make it particularly suitable for liquid biopsy applications. The ability to detect and monitor both viral and tumor-derived biomarkers provides multiple layers of information that can guide clinical decision-making throughout the patient journey.

Technical advances in molecular detection methods, coupled with an improved understanding of biomarker biology, have enhanced the sensitivity and specificity of liquid biopsy testing. Standardization efforts and quality control measures have improved result reliability and reproducibility across different clinical settings. However, challenges remain in optimizing pre-analytical variables and establishing universal testing protocols.

The clinical utility of liquid biopsy in HPV-associated head and neck cancer has been demonstrated across multiple applications, from early detection to treatment monitoring and surveillance. Cost-effectiveness analyses suggest favorable outcomes when liquid biopsy is integrated into standard care pathways, particularly considering the potential for earlier intervention in cases of recurrence.

Implementation considerations, including laboratory infrastructure, personnel training, and regulatory compliance, continue to shape the adoption of liquid biopsy testing. Successful integration requires careful attention to workflow optimization, quality management, and result reporting systems. Educational initiatives targeting healthcare providers and patients are essential for appropriate test utilization.

Looking ahead, emerging technologies and novel biomarker discoveries promise to further expand the capabilities of liquid biopsy. Artificial intelligence and advanced computational methods are improving data analysis and interpretation, while point-of-care testing platforms may enable more rapid clinical decision-making. The evolution of personalized testing strategies and integrated diagnostic approaches suggests an increasingly important role for liquid biopsy in precision oncology.

The field stands at an exciting junction, with the potential to significantly impact patient care through earlier detection, more precise monitoring, and better-informed treatment decisions. Continued research, technological innovation, and clinical validation will be essential to fully realize the promise of liquid biopsy in HPV-associated head and neck cancer. Future research should aim to harmonize laboratory protocols, develop cost-effective testing algorithms, and integrate quality control measures specific to liquid biopsy for broader clinical implementation.

## Figures and Tables

**Table 1:** Comparative analysis of different diagnostic methods based on key parameters such as sensitivity, specificity, cost, turnaround time, clinical use, and economic feasibility.

Method	Sensitivity	Specificity	Cost	Turnaround Time	Clinical Use	Economic Feasibility
**qPCR**	75–85%	>90%	Low	24–48 h	Limited due to lower sensitivity	High (widely available, low-cost)
**ddPCR**	>95%	>98%	Medium	24–72 h	Recommended for real-time monitoring	Cost-effective for clinical use
**NGS (hybrid capture)**	>99%	97%	Very high	7–10 days	Comprehensive profiling, mutation analysis	Expensive, requires bioinformatics support
**NGS (amplicon-based)**	95%	96%	High	5–7 days	Targeted mutation detection	Costly but clinically relevant for resistance monitoring

## Data Availability

Data are contained within the article.
